# 

**Published:** 1893-06

**Authors:** 


CONTENTS.

VOL. XL. No. 6.

| | PAGE. |
|---|---|
| Popular Fallacies | 121 |
| What is Cholera? | 124 |
| Physiological Basis of Labor | 128 |
| A Life Saving Thought | 131 |
| Behind the Counter | 132 |
| Draughts and Deaths | 134 |
| Infectious Diseases | 136 |
| Cold Bathing | 137 |
| Whooping Cough | 138 |
| Hot Milk as a Stimulant | 138 |
| Shell-fish for Invalids | 138 |
| Effect of Environment | 139 |
| Meat Harmful in Summer | 139 |
| Summer Clothing | 140 |
| For the Complexion | 140 |
| Editor's Outlook | 140 |
| Literary | 142 |
| Recreation Bureau Notes | 143 |

